# The state of nutrition care in outpatient hemodialysis settings in Malaysia: a nationwide survey

**DOI:** 10.1186/s12913-018-3702-9

**Published:** 2018-12-04

**Authors:** Ban-Hock Khor, Karuthan Chinna, Abdul Halim Abdul Gafor, Zaki Morad, Ghazali Ahmad, Sunita Bavanandam, Ravindran Visvanathan, Rosnawati Yahya, Bak-Leong Goh, Boon-Cheak Bee, Tilakavati Karupaiah

**Affiliations:** 1 Dietetics Program, Faculty of Health Sciences, Universiti Kebangsaan Malaysia, 50300 Kuala Lumpur, Malaysia; 2 School of Medicine, Faculty of Health and Medical Sciences, Taylor’s University, 47500 Subang Jaya, Malaysia; 3 Department of Medicine, Faculty of Medicine, Universiti Kebangsaan Malaysia Medical Center, 56000 Kuala Lumpur, Malaysia; 4 National Kidney Foundation of Malaysia, Jalan 14/29, 46100 Petaling Jaya, Selangor Malaysia; 5 Department of Nephrology, Kuala Lumpur Hospital, Jalan Pahang, 53000 Kuala Lumpur, Malaysia; 6 Department of Nephrology, Serdang Hospital, Jalan Puchong, 43000 Kajang, Selangor Malaysia; 7 Department of Nephrology, Selayang Hospital, Lebuh Raya Selayang-Kepong, 68100 Batu Caves, Selangor Malaysia; 8 School of BioSciences, Faculty of Health and Medical Sciences, Taylor’s University, 1, Jalan Taylors, 47500 Subang Jaya, Malaysia

**Keywords:** Dietitian, Hemodialysis, Nutrition practices, Survey, Oral nutrition supplement, In-center meals

## Abstract

**Background:**

This study aimed to assess the situational capacity for nutrition care delivery in the outpatient hemodialysis (HD) setting in Malaysia by evaluating dietitian accessibility, nutrition practices and patients’ outcomes.

**Methods:**

A 17-item questionnaire was developed to assess nutrition practices and administered to dialysis managers of 150 HD centers, identified through the National Renal Registry. Nutritional outcomes of 4362 patients enabled crosscutting comparisons as per dietitian accessibility and center sector.

**Results:**

Dedicated dietitian (18%) and visiting/shared dietitian (14.7%) service availability was limited, with greatest accessibility at government centers (82.4%) > non-governmental organization (NGO) centers (26.7%) > private centers (15.1%). Nutritional monitoring varied across HD centers as per albumin (100%) > normalized protein catabolic rate (32.7%) > body mass index (BMI, 30.7%) > dietary intake (6.0%). Both sector and dietitian accessibility was not associated with achieving albumin ≥40 g/L. However, NGO centers were 36% more likely (*p* = 0.030) to achieve pre-dialysis serum creatinine ≥884 μmol/L compared to government centers, whilst centers with dedicated dietitian service were 29% less likely (*p* = 0.017) to achieve pre-dialysis serum creatinine ≥884 μmol/L. In terms of BMI, private centers were 32% more likely (*p* = 0.022) to achieve BMI ≥ 25.0 kg/m^2^ compared to government centers. Private centers were 62% less likely (*p* <  0.001) while NGO centers were 56% less likely (*p* <  0.001) to achieve serum phosphorus control compared to government centers. Patients from centers with a shared/visiting dietitian had 35% lower probability (*p* <  0.001) to achieve serum phosphorus levels below 1.78 mmol/L compared to centers without access to a dietitian.

**Conclusions:**

There were clear discrepancies in nutritional care in Malaysian HD centers. Changes in stakeholder policy are required to ensure that dietitian service is available in Malaysian HD centers.

**Electronic supplementary material:**

The online version of this article (10.1186/s12913-018-3702-9) contains supplementary material, which is available to authorized users.

## Background

Malaysia is an upper middle-income country in Asia [1], where hemodialysis (HD) forms the main choice of renal replacement therapy for patients with end stage kidney disease compared to peritoneal dialysis and kidney transplant [2]. About 33,456 patients are on HD treatment as reported in 2015 with the proportion of delivery by sector changing dramatically over time [2]. The scenario of dialysis treatment has shifted from government-only providers to burden sharing with non-governmental organization (NGO) not for profit centers and more recently private centers have risen to become the largest provider in Malaysia [2].

Although HD treatment is life saving, this population is prone to multiple co-morbidities such as protein energy wasting (PEW), fluid and electrolytes imbalance, mineral bone disorders, and anemia due to dialysis and uremic-induced metabolic disruptions [3]. These co-morbidities are potentially treated by medical nutrition therapy provided by dietitians practicing in nephrology care. Ideally the components of this therapy are implemented as per the standardized nutrition care process to ensure optimal nutrition outcomes [4]. Specifically, nutrition assessment is the first critical step of the nutrition care process as it calls for identification of nutrition-related issues of HD patients leading to formulation of the nutrition diagnosis, which then sets the stage for nutrition intervention [5].

The optimal ratio of dietitian to patients in nephrology care has been suggested to be 1:100 patients, not exceeding 150 [4]. However, dietitian services have been observed to be limited in Malaysia and nutritional management was primarily carried out by physicians and nurses [6]. Concurrent with this limitation, the National Renal Registry (NRR) of Malaysia has been annually reporting on nutritional status as assessed by body mass index (BMI) and serum albumin from 2003 [7]. Noticeably, based on these two parameters alone, annual malnutrition reportage for Malaysian HD patients with 10-year trends from 2006 to 2015 have shown an increasing trend from 46 to 62% with serum albumin < 4.0 g/L, in contrast to a decreasing trend of BMI < 25 kg/m^2^ from 71 to 61% [8]. This limited data does not identify PEW, a condition characterized by loss of body protein muscle mass and fuel reserves, and which is suggested to be the core of malnutrition linked to mortality [9, 10]. Recently, 38.5% of Malaysian HD patients were identified with PEW using the diagnostic criteria of the International Society of Renal Nutrition and Metabolism [11]. Given this background, we felt it was critical to examine the current state of dietitian accessibility and nutrition practices in Malaysian HD centers.

## Methods

### Study design and sample

This cross-sectional study involved HD centers from government, private and NGO sectors. Through random stratified sampling, 153 HD centers were selected from 667 HD centers registered with the Malaysia NRR for the year 2015. This sampling ensured adequate representation of all states within Malaysia. Data collection was conducted via telephone interviews with the dialysis managers of participating HD centers from November 2015 to March 2016. In addition, we captured annual patient data of these centers for the year 2015 from the NRR database. The protocol for this study received ethical approval from the Research Ethics Committee, National University of Malaysia (NN-079-2015) and Medical Research Ethic Committee, Ministry of Health, Malaysia (NMRR-15-1245-27039).

### Questionnaire development

A 17-item questionnaire was designed to assess nutrition care provision at HD centers (Additional file 1). Three renal dietitians and a senior dialysis nurse reviewed and established content validity for the questionnaire. The questionnaire consisted of 4 sections:

- Section 1: Characteristics of the HD center such as sector, number of patients, and presence of a dietitian.
- Section 2: Nutrition parameters routinely monitored for HD patients and healthcare professionals involved in delivery of nutrition education.
- Section 3: Recommendation, indications, contraindications, and provision of renal specific oral nutrition supplements (ONS).
- Section 4: Practice of eating and provision of in-center meal during dialysis.

### In-center meals provision

In-center meals provided during the dialysis were examined for nutritional composition. Personal communications with dialysis managers and dietitians from HD centers providing in-center full meals were established to enable access to the menu and portion sizes of food served. Nutrient analysis was carried out using software Nutritionist Pro™ 2.2.16 software (First DataBank Inc., 2004) with reference to the Malaysian [12] and Singapore Food Composition [13] databases.

### Statistical analyses

Continuous variables with normal distribution were presented as means ± standard deviations while skewed continuous variables were presented as median (interquartile range). Categorical variables were presented as frequency (percentage). Chi-square was used to identify associations between categorical variables. Independent *t*-test and one-way ANOVA were used to compare means of continuous variables for groups identified by sector and dietitian accessibility. Bonferroni post hoc test allowed for paired comparisons between groups. Kruskal-Walis test examined for significance of non-normal distributions of continuous variables with Dunn’s comparison used for post hoc analysis. Pearson’s Chi Square was used to assess relationships between two categorical variables. Univariate analysis was used to evaluate continuous variables by incorporating covariates with Bonferroni post hoc test for pairwise comparison. Binary logistic regression analysis identified dietitian’s accessibility and center sector associated with nutrition parameters achieving the Kidney Disease Outcomes Quality Initiative (KDOQI) recommendations [14]. Statistical analyses were computed using the IBM SPSS version 26.0 (IBM SPSS Statistics Inc. Chicago IL. USA) and statistical significance level was set as *p* <  0.05.

## Results

### HD center distribution and dietitian access

Of 153 HD centers contacted, 3 centers refused to participate, leaving only 150 HD centers for respondent inclusion. The characteristics of these HD centers are summarized in Table 1. By sector distribution, private centers (57.3%) dominated over government (22.7%) and NGO (20.0%) centers. Regionally the HD center distribution was as per the Central region (28.0%) > Northern region (23.3%) > East Coast (21.3%) > Borneo (14.0%) > Southern region (13.0%). Majority reported lack of dietitians with only 18.0% reporting access to a dedicated dietitian and 14.7% having access to a visiting or shared dietitian. Most government centers had access to either dedicated, visiting or shared dietitians contrasting with poor access to a dietitian in both private (84.9%) and NGO (73.3%) HD centers. In particular, no NGO center had access to dedicated dietitian service. Lack of dietitian service was noticeable for HD centers in the Southern region (90%) compared to other regions (ranged from 59.4 to 68.6%). Number of patients per center with a dedicated dietitian differed significantly from centers without a dietitian (71.4 ± 37.8 vs. 49.5 ± 30.4 respectively, *p* = 0.005).

**Table 1:** Characteristics of participating HD centers

		Dietitian Accessibility		
	All (*n* = 150)	Not available (*n* = 101)	Dedicated dietitian (*n* = 27)	Shared/ visiting dietitian (*n* = 22)
*By Sector*				
Government	34 (22.7%)	6 (17.6%)	19 (55.9%)	9 (26.5%)
NGO	30 (20.0%)	22 (73.3%)	–	8 (26.7%)
Private	86 (57.3%)	73 (84.9%)	8 (9.3%)	5 (5.8%)
*By Region*				
Central	42 (28.0%)	27 (64.3%)	10 (23.8%)	5 (11.9%)
East Coast	32 (21.3%)	19 (59.4%)	6 (18.8%)	7 (21.9%)
Northern	35 (23.3%)	24 (68.6%)	5 (14.3%)	6 (17.1%)
Southern	20 (13.3%)	18 (90.0%)	2 (10.0%)	–
Borneo	21 (14.0%)	13 (62.0%)	4 (19.0%)	4 (19.9%)
*Number of patients/center* ^a^	54.5 ± 32.1	49.5 ± 30.4	71.4 ± 37.8	56.2 ± 25.6
< 50	80 (53.7%)	63 (78.8%)	7 (8.8%)	10 (12.5%)
50–100	55 (36.9%)	29 (52.7%)	15 (27.3%)	11 (20.0%)
> 100	14 (9.4%)	8 (57.1%)	5 (35.7%)	1 (7.1%)

^a^*p* <  0.05 using one-way ANOVA test and Bonferroni post hoc test indicated significant difference between dedicated dietitian vs. no dietitian

Data is presented as either *n* (%) or mean ± standard deviation

Abbreviation: *NGO* non-governmental organization

### Nutrition monitoring and education

Nutrition monitoring, nutrition education, use of renal specific ONS and provision of in-center meals were nutrition care domains assessed in this situational analysis (Table 2). Serum albumin was used by all HD centers for nutrition monitoring, followed by normalized protein catabolic rate (*n*PCR), BMI and dietary assessment. Neither sector distribution of HD centers nor dietitian access significantly correlated with nutrition monitoring (*p* > 0.05). None of the HD centers reported using any of the available nutrition-screening tools such as Subjective Global Assessment, Malnutrition Inflammation Score or Dialysis Malnutrition Score.

**Table 2:** Comparison of nutrition practices by sector and dietitian accessibility across hemodialysis centers

Nutrition Practices	All (*n* = 150)	Sector			Dietitian Accessibility		
		Government (*n* = 34)	Private (*n* = 86)	NGO (*n* = 30)	Not available (*n* = 101)	Dedicated dietitian (*n* = 27)	Shared/ visiting dietitian (*n* = 22)
*Nutrition Monitoring*							
BMI	46 (30.7%)	15 (44.1%)	22 (25.6%)	9 (30.0%)	26 (25.7%)	12 (44.4%)	8 (36.4%)
Albumin	150 (100%)	34 (100%)	86 (100%)	30 (100%)	101 (100%)	27 (100%)	22 (100%)
*n*PCR	49 (32.7%)	11 (32.4%)	28 (32.6%)	10 (33.3%)	34 (33.7%)	9 (33.3%)	6 (27.3%)
Dietary	9 (6.0%)	1 (2.9%)	5 (5.8%)	3 (10.0%)	5 (5.0%)	2 (7.4%)	2 (9.1%)
Nutrition Screening Tool	nil	nil	nil	nil	nil	nil	nil
*Who delivers Nutrition Education*?							
Dietitian	48 (32.0%)	27 (79.4%)	13 (15.1%)	8 (26.7%)	nil	27 (100%)	22 (100%)
Medical doctor	149 (99.3%)	34 (100%)	86 (100%)	29 (96.7%)	100 (99.0%)	27 (100%)	22 (100%)
Nurse	150 (100%)	34 (100%)	86 (100%)	30 (100%)	101 (100%)	27 (100%)	22 (100%)
*How is Nutrition Education delivered?*							
Individual	103 (68.7%)	22 (64.7%)	62 (72.1%)	19 (63.3%)	75 (74.3%)	17 (63.0%)	11 (50.0%)
Group sessions	13 (8.7%)	2 (5.9%)	10 (11.6%)	1 (3.3%)	9 (8.9%)	1 (3.7%)	3 (13.6%)
Both	34 (22.7%)	10 (29.4%)	14 (16.3%)	10 (33.3%)	17 (16.8%)	9 (33.3%)	8 (36.4%)
*Recommendation for Renal Specific ONS*							
Yes	103 (68.7%)	15 (44.1%)	67 (77.9%)	21 (70.0%)	71 (70.3%)	19 (70.4%)	13 (59.1%)
Free of charge	10 (9.7%)	7 (46.7%)	nil	3 (14.3%)	1 (1.4%)	6 (31.6%)	3 (23.1%)
Buy from dialysis center	31 (30.1%)	1 (6.7%)	21 (31.3%)	9 (42.9%)	19 (26.8%)	6 (31.6%)	6 (46.2%)
Buy from outside	62 (60.2%)	7 (46.7%)	46 (68.7%)	9 (42.9%)	51 (71.8%)	7 (36.8%)	4 (30.8%)
*Provision of In-center Meals*							
Yes	92 (61.3%)	23 (67.6%)	63 (73.3%)	7 (23.3%)	61 (60.4%)	22 (81.5%)	10 (45.5%)
Full meal	21 (22.8%)	15 (65.2%)	6 (9.5%)	nil	6 (9.8%)	11 (50.0%)	4 (40.0%)
Light meal	71 (77.2%)	8 (34.8%)	57 (90.5%)	7 (100%)	55 (90.2%)	11 (50.0%)	6 (60.0%)

Note: Data is reported as *n* (%)

Abbreviation: *BMI* body mass index, *NGO* non-governmental organization, *nPCR* normalized protein catabolic rate, *ONS* oral nutrition supplement

Nurses (100%) and physicians (99.3%) were reported to be regularly involved in providing nutrition education in HD centers compared to dietitians (32.0%). The sector of HD centers significantly correlated in terms of nutrition education provided by a dietitian (χ^2^ = 43.011, *p* <  0.001) and were more common in government HD centers (79.4%) compared to private (15.1%) or NGO (26.7%) centers. Nutrition education was primarily provided individually rather than via group sessions or both in all centers. Some noteworthy comments by dialysis managers on nutrition education were:

- “*Our medical doctors in charge will provide nutrition education during 3-monthly routine follow-up based on patients’ blood investigations*” (NGO center in East Coast, center code: 59)
- “*We only provide nutrition pamphlets produced by drug companies to patients*” (private center in Central region, center code: 25)
- “*Nutrition education will be given as part of the overall health education module given to patients*” (private center in East Coast, center code 114)
- “*Our dedicated nurses have undergone training on nutrition management of dialysis patients and we have regular meetings to discuss about nutrition issues of patients*” (NGO center in Southern region, center code: 45)

### Renal specific ONS

**Fig. 1:**
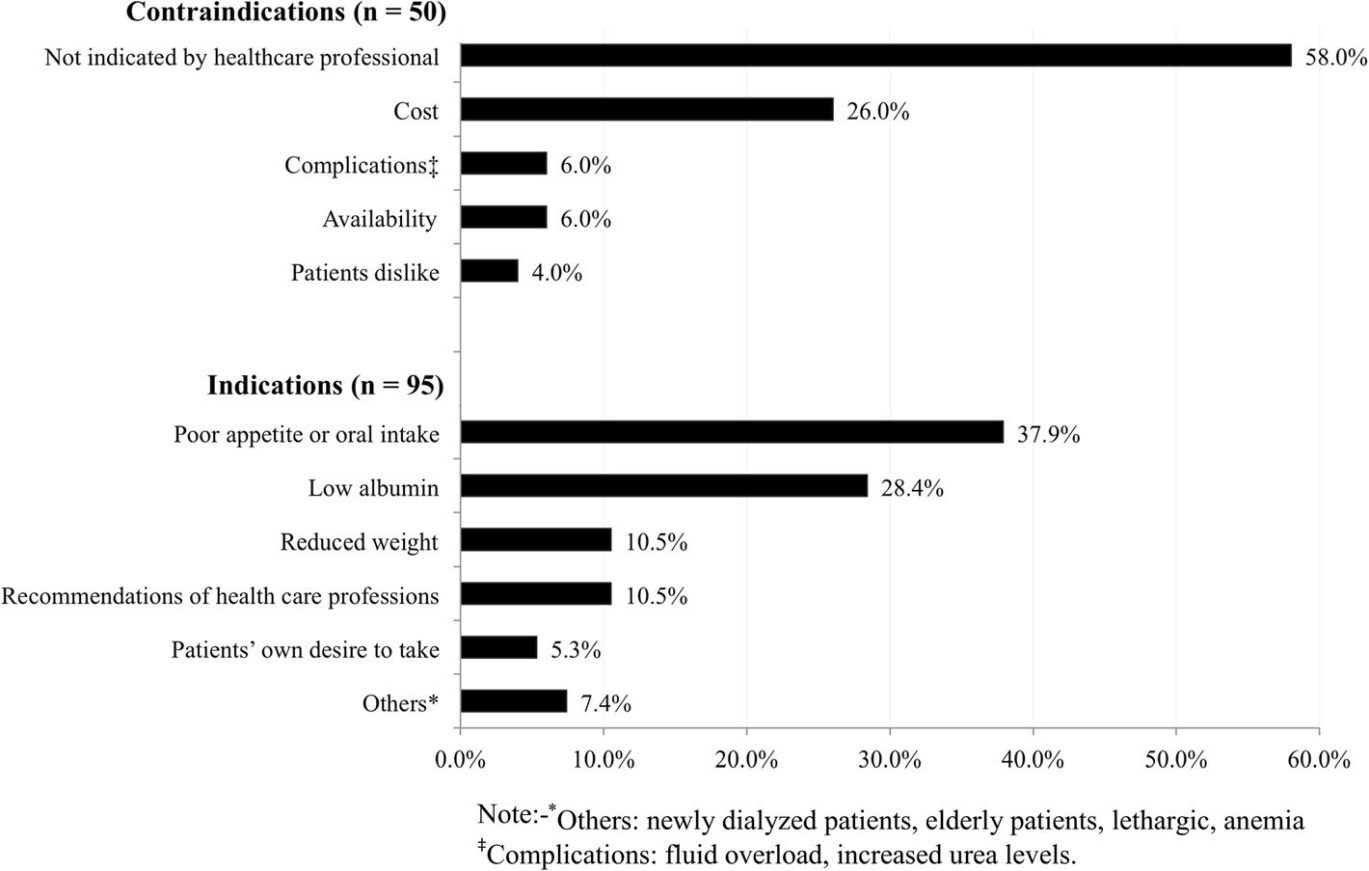
Contraindications and Indications for Use of Renal Specific ONS

Recommending use of renal specific ONS was reported by 68.7% of HD centers, which significantly correlated with HD sector (χ^2^ = 12.961, *p* = 0.002). It was more common in private (77.9%) and NGO (70.0%) centers compared to government (44.1%) centers. However, only 9.7% of HD centers provided ONS at no cost to patients but none by private centers. Figure 1 lists the indications and contraindications by dialysis managers for recommending renal specific ONS to patients.

### In-center meals provision

All HD centers allowed patients to eat during treatment, but 10 (6.7%) centers advised against heavy meal consumption. Provision of in-center meals was significantly correlated by sector (χ^2^ = 23.584, *p* <  0.001) and dietitian access (χ^2^ = 8.529, *p* = 0.014), with NGO centers (23.3%) and centers with visiting dietitians (40.9%) having a lower frequency of in-center meals provision (Table 2). Twelve HD centers provided menus of in-center meals served during treatment with menu rotations ranging between 3 to 14 days. The nutrient content (means ± standard deviations) of these in-center meals provided in 12 HD centers were 469.1 ± 108.5 kcal, 57.0 ± 18.8 g carbohydrate, 24.8 ± 8.7 g protein, 15.6 ± 7.3 g fat, 400.0 ± 497.3 mg sodium, 519.0 ± 225.0 mg potassium, 281.9 ± 164.6 mg phosphorus, and 322.1 ± 86.0 ml fluid.

### Nutrition outcomes of patients

Four out of 150 participating centers did not submit patients’ annual data to NRR, and 2679 patients’ annual data from the remaining 146 centers were incomplete, leaving only annual data of 4362 patients available for statistical analysis. Patients’ characteristics and nutrition parameters are summarized in Table 3. Pre-dialysis serum creatinine was significantly lower in centers with access to a dietitian compared to without a dietitian (*p* = 0.004) whereas center sector variation was not a factor (*p* = 0.431). On the other hand, BMI significantly differed by sector, with higher BMI values prevalent in patients from private centers compared to government (24.6 kg/m^2^ vs. 23.5 kg/m^2^, *p* <  0.001) and NGO centers (24.6 kg/m^2^ vs. 23.8 kg/m^2^, *p* = 0.003). Serum albumin levels also significantly differed by sector with patients from private HD centers having significantly lower serum albumin levels compared to patients from NGO centers (38.6 g/L vs. 39.4 g/L, *p* <  0.001). Serum phosphorus levels were also significantly different by sector and dietitian accessibility. Serum phosphorus levels were lowest in patients from government centers, followed by NGO and private centers (1.62 mmol/L vs. 1.78 mmol/L vs. 1.84 mmol/L). Contrarily, significant higher phosphorus levels were observed in patients with access to a shared or visiting dietitian compared to a dedicated dietitian (1.79 mmol/L vs. 1.74 mmol/L, *p* <  0.001) or no dietitian (1.79 mmol/L vs. 1.74 mmol/L, *p* <  0.001).

**Table 3:** Comparisons of patients’ characteristics and nutrition parameters by sector and dietitian accessibility in Malaysian hemodialysis centers

Patient Characteristics and Nutrition Parameters	All (*n* = 4362)	Sector			*p* value	Dietitian Accessibility			*p* value
		Government (*n* = 1072)	Private (*n* = 1949)	NGO (*n* = 1341)		Not available (*n* = 2618)	Dedicated dietitian (*n* = 934)	Shared/ visiting dietitian (*n* = 810)	
Age (year)	55.0 ± 13.53	52.2 ± 14.7	56.8 ± 12.6	54.6 ± 13.5	< 0.001^a^	55.0 ± 13.00	54.6 ± 14.8	55.5 ± 13.70	0.395
Gender (Male)	2484 (59.6%)	589 (54.9%)	1103 (56.9%)	792 (59.1%)	0.117	1491 (57.0%)	531 (56.9%)	462 (57.0%)	0.997
Dialysis vintage (month)	43.0 (59)	53.5 (78)	34.0 (45)	50.0 (65)	< 0.001^b^	38.0 (51)	50.5 (78)	51.0 (62)	< 0.001^b^
Pre-dialysis creatinine (μmol/L)	840 ± 239	813 (796–831)	822 (813–840)	831 (813–849)	0.431	840 (831–849)	804 (787–822)	822 (804–840)	0.006^c^
BMI (kg/m^2^)	24.1 ± 4.7	23.5 (23.2–23.8)	24.6 (24.2–24.9)	23.8 (23.5–24.2)	< 0.001^d^	23.8 (23.5–24.0)	23.9 (23.5–24.3)	24.2 (23.8–24.6)	0.187
Albumin (g/L)	38.8 ± 4.3	39.1 (38.8–39.4)	38.6 (38.3–38.9)	39.4 (39.1–39.7)	< 0.001^e^	38.9 (38.6–39.1)	39.2 (38.8–39.6)	39.0 (38.6–39.3)	0.349
Phosphorus (mmol/L)	1.75 ± 0.45	1.62 (1.58–1.65)	1.84 (1.80–1.87)	1.78 (1.74–1.81)	< 0.001^f^	1.70 (1.67–1.72)	1.74 (1.70–1.78)	1.79 (1.76–1.83)	< 0.001^g^

Univariate analysis adjusted for age, gender, dialysis vintage, Kt/V, number of patients in the center, center sector, and dietitian accessibility was used to compare nutrition parameters. Data of nutrition parameters is presented as adjusted mean (95% confidence interval)

^a^One-way ANOVA Test, Bonferroni post hoc test indicates significant difference government vs. private vs. NGO

^b^Kruskal Wallis Test

^c^Bonferroni post hoc test indicates *p* < 0.05 for pairwise comparison dedicated dietitian vs. no dietitian

^d^Bonferroni post hoc test indicates *p* < 0.05 for pairwise comparison private vs. government and private vs. NGO

^e^Bonferroni post hoc test indicates *p* < 0.05 for pairwise comparison private vs. NGO

^f^Bonferroni post hoc test indicates *p* < 0.05 for all pairwise comparisons

^g^Bonferroni post hoc test indicates *p* < 0.05 for pairwise comparison shared/visiting dietitian vs. no dietitian and shared/visiting dietitian vs. dedicated dietitian

Abbreviations: *BMI* body mass index, *NGO* non-governmental organization

These nutrition parameters were then categorized in accordance with achievement of the KDOQI recommendations [14] namely, pre-dialysis serum creatinine ≥884 μmol/L, BMI ≥ 25.0 kg/m^2^, serum albumin ≥40 g/L, and serum phosphorus ≤1.78 mmol/L (Table 4). Both sector and dietitian accessibility was not associated with achieving albumin ≥40 g/L. However, patients from NGO centers were 36% more likely (95% CI: 1.03, 1.81; *p* = 0.030) to achieve pre-dialysis serum creatinine ≥884 μmol/L compared to patients from government centers, while patients from centers with dedicated dietitian service were 29% less likely (95% CI: 0.54, 0.94; *p* = 0.017) to achieve pre-dialysis serum creatinine ≥884 μmol/L than patients from centers without access dietitian services. In terms of BMI, patients of private center were 32% more likely (95% CI: 1.04, 1.67; *p* = 0.022) to achieve BMI ≥ 25.0 kg/m^2^ compared to government centers, whilst dietitian access was not a related factor (*p* > 0.05). Private patients were 62% less likely (95% CI: 0.30, 0.45; *p* <  0.001) while NGO patients were 56% less likely (95% CI: 0.34, 0.56; *p* <  0.001) to achieve serum phosphorus control compared to government patients. Patients from centers with a shared/visiting dietitian had 35% lower probability (95% CI: 0.53, 0.81; *p* <  0.001) to achieve serum phosphorus levels below 1.78 mmol/L compared to centers without access to a dietitian.

**Table 4:** Adjusted odds ratio by hemodialysis center sector and dietitian accessibility for achieving KDOQI nutritional outcomes

	Serum Creatinine ≥884 μmol/L		BMI ≥ 25 kg/m^2^		Serum Albumin ≥40 g/L		Serum Phosphorus ≤1.78 mmol/L	
	OR_adj_ (95% CI)	*p* value	OR_adj_ (95% CI)	*p* value	OR_adj_ (95% CI)	*p* value	OR_adj_ (95% CI)	*p* value
*HD Sector*								
Private	1.25 (0.96, 1.63)	0.103	1.32 (1.04, 1.67)	0.022	0.97 (0.76, 1.25)	0.124	0.38 (0.30, 0.45)	< 0.001
NGO	1.36 (1.03, 1.81)	0.030	0.95 (0.74, 1.22)	0.688	0.83 (0.66, 1.05)	0.827	0.44 (0.34, 0.56)	< 0.001
Government	1.00		1.00		1.00		1.00	
*Dietitian Accessibility*								
Shared/visiting dietitian	0.85 (0.67, 1.08)	0.074	1.13 (0.91, 1.41)	0.281	1.18 (0.95, 1.46)	0.129	0.65 (0.53, 0.81)	< 0.001
Dedicated dietitian	0.71 (0.54, 0.94)	0.017	0.87 (0.68, 1.12)	0.260	1.05 (0.82, 1.34)	0.689	0.78 (0.61, 1.00)	0.052
No dietitian	1.00		1.00		1.00		1.00	

Note- Binary logistic regression adjusted for age, gender, dialysis vintage, Kt/V, number of patients in the center, center sector, and dietitian accessibility

Abbreviations: *BMI* Body Mass Index, HD *hemodialysis* *NGO* Non-governmental organization, *OR*_*adj*_ *(95% CI)* adjusted odd ratio (95% confidence interval)

## Discussion

This is the first situational analysis carried out on a national scale using randomized sampling of HD centers registered with the NRR, enabling adequate regional representation of the total sampling distribution. The state of renal nutrition practice reported in this study may reflect a similar scenario in other Southeast and South Asian countries, where access to a dietitian may be limited [6]. In Malaysia, overall dietitians’ accessibility (either dedicated, visiting or shared) was available only to 32.7% of HD centers, and this accessibility was restricted to larger urban centers and particularly government centers. In Europe, the availability of dietitians in HD centers ranges between 20% in Spain to 85% in the UK [15] while it is mandatory for dialysis facilities in the United States to have a dietitian member in the multidisciplinary team for patient care [16]. Despite more than 50% of HD delivery in Malaysia being dominated by private centers, patients in these centers had a more limited access to a dietitian compared to patients in government or NGO centers. Several factors may lead to limited dietitian accessibility in private centers: (i) dietitian service is not mandatory for private HD centers in Malaysia, but nephrologists and nurses are considered essential in patient care [17] (ii) patients dialyzed in private centers are paying more for the dialysis treatment, and therefore unable to pay out-of-pocket consultations for the dietitian as this fee is not reimbursed by insurance and (iii) most small scale private centers cannot afford the wage cost for a full time dietitian.

Nutrition assessment is an essential and fundamental component to identify patients with critical nutrition issues such as PEW [18]. As expected, we found nutrition assessment practices were relatively uniform across different HD sectors with reliance on serum albumin as the standard monitoring parameters. A shortcoming in relying on albumin alone to identify malnutrition, without validating with BMI and dietary intake, is the risk of potential false negatives arising from inflammation, fluid overload and infection [19, 20]. Although all centers used serum albumin as a reference nutritional marker, alarmingly only one-third of centers added monitoring data for BMI and *n*PCR. BMI is a simple and inexpensive nutrition assessment tool, which correlates with clinical outcomes in HD patients [21] while *n*PCR is already used as a proxy of estimating dietary protein intake by 38% of HD centers in Europe [15]. We also found that overall, dietary assessment was rarely performed and no HD centers reported using any nutrition-screening tool, irrespective of dietitian accessibility. A thorough dietary assessment is critical as it allows personalized advice to be provided to dialysis patients with various nutrient restrictions [22]. The non-participation of dietitians in performing regular nutrition assessment was observed even in HD centers with dietitian access, as dietitians only ‘take care’ of patients who had been pre-screened and referred by other healthcare professionals. Therefore, it is not surprising that we could not establish any relationship between dietitian accessibility and nutrition assessment practices. Moreover, the absence of a dietitian means lack of expertise and skills in performing certain nutrition assessments such as handgrip strength, skinfold measurement and dietary interviews [23].

The algorithm for nutritional management of PEW patient by International Society of Renal Nutrition and Metabolism recommends optimizing calorie and protein intakes by providing nutritional support [15]. We therefore, also assessed the practice of renal specific ONS use in these centers. Dialysis managers reported indications for products use for HD patients but we found that some of the indications did not fit the criteria for use [24]. Perceptions of dialysis managers were patients were well nourished despite the lack of proper nutrition assessment along with a view of increased cost burden to patient cost burden are likely obstacles hindering the long terms use of ONS to optimizing nutrition for PEW patients. Despite this negativity, a small percentage of HD centers provided the renal specific ONS free of charge to the patients.

Eating during dialysis is common in all Malaysian HD centers, which is also similar to other countries [25]. It has been suggested that eating during dialysis may lead to complications such as postprandial hypotension, gastrointestinal symptoms, choking and reduced treatment efficiency [26]. However, in practice, these issues have not been often observed in our settings. On the contrary, eating during dialysis has been suggested to improve nutritional status, quality of life, and inflammation status as well as to provide teaching opportunities to patients [26].

It has been conclusively shown that dietitians’ care improves nutrition and clinical outcomes of HD patients [27–29]. In addition, dietitians have other important roles in HD centers such as knowledge sharing with co-health care professionals, quality assessment and performance improvement, research, and protocol/algorithm development and monitoring, which may indirectly translate into improved patients’ nutrition outcomes [5]. We attempted to compare the nutrition outcomes between centers with dietitian access and centers without dietitian access but we did not observe favorable nutrition outcomes associated with dietitian accessibility. There are some possible explanations for this observation. In Malaysia, dietitians are not routinely involved in the clinical practice at HD centers and only attend to selected patients based on physician referral. In addition, the norm in Malaysia is dietitians are assigned to cover several areas including the food service, inpatient management, as well as chronic disease management in outpatient settings. The heavy workload imposed on a dietitian may hinder them from proactively attending to all patients in HD centers. In this context, accessibility to a dietitian does not necessarily translate into more contact time with the patient. Therefore, our findings do not refute the importance of dietitians’ involvement in patient care. Instead, it calls for a strategic human resource management to allow dietitians to be more proactive in routine nutrition management in HD centers.

Marginally lower albumin levels were noted in patients from private HD centers compared to NGO centers but all center mean values were still close to the reference target of 40 g/L [14]. Serum phosphorus levels were observed to vary according to HD sector and likely affected by type of phosphorus binder prescription and dialysis regimes [30]. In Malaysia, government HD centers are known to be resourceful in achieving outcome-based goals targeting hyperphosphatemia, and medical teams without dietitians (nephrologists and nurses) are willing to optimize nutrition guidelines (*personal communication from BLG and SB*). Dietitian accessibility as indicated by this survey meant relying on visiting dietitians to optimize phosphorus control was not better than without a dietitian at all. This perhaps can be attributed to visiting dietitians only providing group education to patients rather than individual patient-centered care as well as a lack of follow-up in the Malaysian scenario. In fact, intensive intervention by dietitians is required for effective phosphorus control in the United Kingdom, which is known to have dedicated dietitian services available for renal patient care [31]. Contrarily, nutrition education delivered by dialysis nurses and physicians may also be equally effective in reducing serum phosphorus as reported in China [32]. Tsai et al. [33] have shown that dietary education by dialysis staff alone resulted in reducing serum phosphorus levels of HD patients, but involvement of dietitians provided additional benefits on controlling hyperphosphatemia while Blair et al. [34] did not observe significant difference in serum phosphorus level in HD patients managed by dietitians or non-dietitians (nephrologists and nurses).

Our study had certain strengths and limitations. This was the first study to evaluate nutrition practices in Malaysian HD centers at national level. We collected information from HD centers representative of different sectors and regions. However, this survey relied on information provided by dialysis managers rather than direct evaluation of practices at HD centers. Information on the presence of resident nephrologists, medical treatment details, and dietitians’ contact time and competencies were not collected in this survey, which may affect interpretation of the results. Furthermore the NRR database, from which we retrieved patient data, lacked critical information on socioeconomic status which would have allowed us to adjust for confounders in examining the relationship of nutritional outcomes to accessibility of dietitian. However other confounders such as age, gender, dialysis vintage and adequacy were included in the statistical analysis. Moreover, the temporal relationship between dietitian accessibility and nutrition outcomes could not be confirmed due to the cross-sectional design and potential residual confounding factors. Despite the limitations, we have generated results that identified gaps in the current status of nutrition care practice in Malaysian HD centers, which is relevant to inform on the planning of improvement strategies to mitigate these gaps.

This study’s findings should inform towards a policy agenda that targets improving dialysis care in Malaysia. As private HD centers continue to increase in Malaysia, this sector is expected to become the major provider of HD treatment. However, patients receiving HD treatment from these facilities going by this study are less likely to have access to dietitian services. A policy development by the stakeholder is urgently called for, to ensure adequate dietitian accessibility in renal patient care in this dialysis sector, which is fast growing in Malaysia. Another option to explore would be locum dietitian services towards benefiting dialysis patients. However, a major development should be the advancement in renal dietetic skills relevant to chronic kidney disease patient management, which would allow for competency development and credentialing for dietitians. A critical aspect to consider is that holistic patient-centered care should include strategies to detect, intervene and audit outcomes for dialysis patients with poor nutritional status.

## Conclusion

Variability of nutrition practices was observed in Malaysian HD centers and the standard of existing nutrition care was generally unsatisfactory due to lack of attention to patient-centered approaches such as personalized nutrition intervention as well as implementation of standardized nutrition guidelines. A dedicated dietitian in HD centers may be essential to standardize nutrition practices and improve nutrition care, which is an important aspect of comprehensive treatment provided to HD patients. Interestingly, nutritional outcome disparities are observed in patients from different HD centers sector, which warrant further investigation.

## Additional file

Additional file 1: A survey on nutrition practices in Malaysian hemodialysis centers. (DOCX 17 kb)

## Abbreviations

BMI: Body mass index
HD: Hemodialysis
KDOQI: Kidney Disease Outcomes Quality Initiative
NGO: Non-governmental organization
*n*PCR: Normalized protein catabolic rate
NRR: National Renal Registry
ONS: Oral nutrition supplement
PEW: Protein energy wasting
