# Supplementary material for: The state of nutrition care in outpatient hemodialysis settings in Malaysia: a nationwide survey
Source: BMC Health Serv Res. 2018 Dec 4;18:939. doi: 10.1186/s12913-018-3702-9 (PMC6280465; doi:10.1186/s12913-018-3702-9)
Supplement: Supplementary file 1 — A survey on nutrition practices in Malaysian hemodialysis centers. (DOCX 17 kb) [file 12913_2018_3702_MOESM1_ESM.docx]

**A Survey on Nutrition Practices in Malaysian Hemodialysis Centers**

**Section A: Your Center Characteristics**

1. Type of dialysis center:

☐ Government ☐ Private ☐ Non-Government Organization (NGO)

1. Total number of hemodialysis patients at your dialysis center: ____________________
2. Does the dialysis center have access to a dietitian?

☐ Yes, full time dietitian

☐ Yes, part time/visiting dietitian

☐ No

**Section B: Nutrition Screening & Education**

1. What is/are the nutrition parameter/s monitored routinely for patients at your center? *(more than one answer may be chosen)*

☐ Body Mass Index

☐ Serum albumin

☐ normalized protein catabolic rate (nPCR)

☐ Dietary intake

☐ Subjective Global Assessment

☐ Malnutrition inflammation score

☐ Others (please state: __________________________________________)

☐ Do not monitor any nutritional parameter.

1. At your center, do you provide ongoing nutrition education to patients?

☐ Yes ☐ No (*go to question 11 if the answer is no*)

1. Who provide/s the nutrition education to your patients? *(more than one answer may be chosen)*

☐ Doctors

☐ Dietitians

☐ Nurses

☐ Medical assistants

☐ Pharmacists

☐ Others (please state: _____________________________________________)

1. What form of education material/s is/are used for the nutrition education? *(more than one answer may be chosen)*

☐ Posters

☐ Flipchart

☐ Pamphlet

☐ Booklet

☐ Multimedia (e.g. video, software or apps)

☐ Others (please state: __________________________________________)

1. How frequently is nutrition education provided to your patients?

☐ Regular basic (please state frequency: _____times in a year)

☐ As per required/referral by doctors

☐ Others (please state: __________________________________________)

1. How is the nutrition counseling delivered to your patients?

☐ Individual counseling

☐ Group counseling

☐ Both

**Section C: Renal Specific Oral Nutrition Supplement (ONS)**

1. Are your patients recommended any renal specific oral nutrition supplement (ONS) on a regular basis?

☐ Yes ☐ No (go to question 14)

1. What is/are the indications for recommending renal specific ONS to your patients?

____________________________________

1. Who recommends the use of ONS to your patients? *(more than one answer may be chosen)*

☐ Doctors

☐ Dietitians

☐ Nurses

☐ Medical assistants

☐ Pharmacists

☐ Patients themselves/their family members

☐ Others (please state: _____________________________________________)

1. Does your center provide ONS to the patients?

☐ Yes, free of charge.

☐ Yes, with patients buying.

☐ No, patients buy outside.

1. Why is renal specific ONS NOT recommended to your patients who need them?

____________________________________

**Section D: In-center Meal Provision**

1. Are your patients allowed to eat during their dialysis session?

☐ Yes ☐ No

1. Does your center provide meals to the patients?

☐ Yes ☐ No (end of the question)

1. What kind of meals are provided to the patients?

☐ Full meal ☐ Light meal
